# Anomalies in alloxan-induced diabetic model: It is better to standardize it first

**DOI:** 10.4103/0253-7613.75684

**Published:** 2011-02

**Authors:** Dinesh K. Jain, Raj Kumar Arya

**Affiliations:** Department of Pharmacology, G.R. Medical College, Gwalior, Madhya Pradesh - 474 001, India

Sir,

Antidiabetic effect of more than 500 plants has been proved in alloxan-induced diabetic animal models but further medical research does not support their effectiveness. We also have been not able to get a single effective antidiabetic molecule from them. It is also understood that alloxan-induced diabetic model does not exactly simulate the human type 2 diabetes mellitus. Some herbomineral products, which have antidiabetic effect in animal models, were not effective in humans as an antidiabetic. Shilajeet and *Gymmema sylvestre* have antidiabetic effects in alloxan-induced animal model but these herbs have no antidiabetic effects when studied in man. There are inconsistencies in doses of drugs, routes of administration, duration and severity of diabetes and methodology in alloxan-induced diabetic models, which make its accuracy controversial. The pharmacokinetic and pharmacodynamic profile of alloxan is ignored by researchers working on these aspects. We wish to make the following observations in this regard.

1. Alloxan is very rapidly destroyed in the body. Its half-life is less than 1 minute. Hence, its action is most unpredictable unless it is administered by rapid intravenous injection. Slow rate of intravenous injection reduces the efficacy of the agent.[1] But in alloxan-induced models, researchers usually use intraperitoneal or other routes, which makes it less capable to induce diabetes of such duration as required for study. During intraperitoneal, intramuscular and subcutaneous routes of administration, onset of alloxan action would be delayed. Will it not be sufficient to destroy alloxan in body? Will delayed onset of action not make it less efficient in producing diabetes in animals?
2. Very young animals have a high resistance to the diabetogenic effect of alloxan.[2]
3. Damage to the renal tubules by alloxan also alters blood glucose level which may also influence the results of study.
4. The susceptibility to both toxic and diabetogenic doses varies widely not only in different species but also among animals of the same species.[1]
5. Rats were more sensitive to a constant dose of alloxan after a fore period on a high fat diet and less sensitive after a high carbohydrate and protein diet.[3]
6. The spontaneous recovery is particularly evident in rats and mice, in which the majority of animals become normoglycemic. The recovery from the diabetes is believed to be the consequence of either a multiplication of β cells from the duct epithelium or the exocrine portion of the pancreas.[4] This is the reason why mild diabetes produced by alloxan recovers spontaneously in rats.
7. Lower doses of alloxan (90-140 mg/kg, i.p.) can result in auto-reversion to normal state. It has been found that in many research studies, the dose of alloxan used to induce diabetes was suboptimal. In these studies, it was found that alloxan induces very mild diabetes. However, in these animals, revert back to the blood glucose values reverted back to normal in a week.
8. β cells of islet of Langerhans in rats show regenerative capacity.[5] Thus, the illusion of antidiabetic effect of experimental product is produced.
9. Production of diabetes also depends upon the speed of the intravenous injections at a given dose level, the slower injection rates causing less effect.[6]
10. Human islet tissue is exceedingly resistant to the degenerative effects of alloxan. Alloxan diabetes differs strikingly from other types of experimental diabetes in the course and nature of the lesions which are developed. As yet, there is no evidence that alloxan-induced diabetes mellitus is related to human diabetes mellitus.[7]

Hence, there are many inconsistencies and anomalies in alloxan-induced diabetic models. By analyzing the above observations and studies mentioned by many authors, it indicates that alloxan-induced diabetic model is a doubtful model for antidiabetic studies. Hence, further studies are needed to evaluate ideal antidiabetic model. Only then, we can develop new effective antidiabetic molecules.
